# Pharmacological enhancement of exposure-based treatment in PTSD: a qualitative review

**DOI:** 10.3402/ejpt.v4i0.21626

**Published:** 2013-10-17

**Authors:** Rianne A. de Kleine, Barbara O. Rothbaum, Agnes van Minnen

**Affiliations:** 1 Behavioral Science Institute, Radboud University Nijmegen, Nijmegen, the Netherlands; 2 Overwaal, Centre for Anxiety Disorders (Pro Persona), Nijmegen, the Netherlands; 3 NijCare, Nijmegen, the Netherlands; 4 Department of Psychiatry and Behavioral Sciences, Emory University School of Medicine, Atlanta, GA, USA

**Keywords:** Posttraumatic stress disorder, cognitive enhancers, treatment efficacy, exposure therapy

## Abstract

There is a good amount of evidence that exposure therapy is an effective treatment for posttraumatic stress disorder (PTSD). Notwithstanding its efficacy, there is room for improvement, since a large proportion of patients does not benefit from treatment. Recently, an interesting new direction in the improvement of exposure therapy efficacy for PTSD emerged. Basic research found evidence of the pharmacological enhancement of the underlying learning and memory processes of exposure therapy. The current review aims to give an overview of clinical studies on pharmacological enhancement of exposure-based treatment for PTSD. The working mechanisms, efficacy studies in PTSD patients, and clinical utility of four different pharmacological enhancers will be discussed: d-cycloserine, MDMA, hydrocortisone, and propranolol.

There is a good amount of evidence that (prolonged) exposure therapy is an effective treatment for posttraumatic stress disorder (PTSD) (Powers, Halpern, Ferenschak, Gillihan, & Foa, 2010). It is a first-line treatment recommended in guidelines worldwide. Nevertheless, not all patients benefit from exposure therapy. Clinical trials have shown that approximately 50% of patients lose their PTSD diagnosis after exposure therapy and that the proportion of patients achieving complete remission is even smaller (Bradley, Greene, Russ, Dutra, & Westen, 2005; Schnurr et al., 2007).

In an attempt to improve treatment efficacy, some researchers added other psychological interventions to exposure therapy, such as cognitive restructuring (Foa et al., 2005; Resick et al., 2008) or imaginal rescripting (Arntz, Tiesema, & Kindt, 2007). Although some studies found support for beneficial effects, overall the effect sizes did not exceed those of stand-alone exposure therapy in a clinically significant way (see for review: Kehle-Forbes et al., 2012).

Another way to improve treatment efficacy that is commonly seen in clinical care is the combination of exposure therapy and pharmacological treatment such as antidepressant medication. However, controlled studies investigating the efficacy of this combined treatment strategy are scarce. Rothbaum et al. (2006) examined the effect of adding prolonged exposure (PE; Foa & Rothbaum, 1998) for SSRI non-responders. PTSD patients were provided with 10 weeks of open-label sertraline and those who did not remit were then randomized to either receive five additional weeks of sertraline alone or with 10 sessions of twice weekly PE. Results show that the addition of 10 sessions of PE led to increased treatment gains but only for patients who showed a partial response to phase I sertraline treatment. PE augmentation was associated with lower PTSD severity score, more remitters at 6 month follow-up, and maintenance of treatment gains. In an almost mirror design, no beneficial effects were found for paroxetine enhancement when given in addition to PE to exposure refractory patients (Simon et al., 2008). In contrast, however, Schneier and colleagues (2012) found that when the combination of exposure therapy and paroxetine (an SSRI) was provided from the beginning of treatment, it was more effective than exposure therapy plus placebo, implying additive benefits. However, the additive benefits disappeared by follow-up. Even though initial treatment with exposure therapy and paroxetine may lead to good clinical outcome, there are also some important disadvantages of this combination strategy, such as adverse events of medication, higher treatment costs, lower treatment acceptability and the risk of relapse after medication discontinuation, as was shown with SSRI as a stand-alone treatment for PTSD (Davidson et al., 2001; Farach et al., 2012).

Recently, an interesting new direction in the improvement of exposure therapy efficacy for PTSD emerged. Basic research in animals found evidence of the pharmacological enhancement of the underlying learning and memory processes of exposure therapy: extinction learning and reconsolidation (Debiec & Ledoux, 2004; Walker, Ressler, Lu, & Davis, 2002). Extinction learning refers to the process wherein a conditioned stimulus (CS; i.e., a trauma reminder) is repeatedly presented in absence of the unconditioned stimulus (US; i.e., the traumatic experience) thereby leading to reduction of the conditioned response (CR; i.e., fear). It is believed that with extinction learning a new association (CS-noUS) is formed and consolidated, while the original fear-memory stays intact (Bouton, 1993; LeDoux, 1995). In contrast, reconsolidation might change the original fear memory. Reconsolidation refers to the process wherein a previously consolidated memory (i.e., the fear memory), enters a labile state upon its retrieval, in which it might be susceptible to change. Even though it is not perfectly understood if and how extinction and reconsolidation inter-relate during exposure therapy (Kindt & Soeter, 2013), both seem to be underlying its efficacy.

Findings in basic animal research that these memory and learning processes can be pharmacologically targeted have been translated to studies in clinical populations (f.i. Ressler et al., 2004). In several anxiety disorders it has been examined whether different pharmacological agents, often referred to as *cognitive enhancers*, can optimize exposure treatment efficacy. This new line of pharmacological treatment enhancement can be distinguished from traditional pharmacotherapy in the following ways: (1) the pharmacological agent is always given in supplementary fashion to exposure by administration either shortly before or after an exposure session; (2) the enhancer is not expected to positively affect treatment outcome as such, but to do so solely by augmentation of exposure effects.

The aim of the current review is to give an overview of clinical studies on pharmacological enhancement of exposure-based treatment for PTSD. Thus far, reviews have focused mainly on fundamental research or specific pharmacological backgrounds (Choi, Rothbaum, Gerardi, & Ressler, 2010; Dunlop, Mansson, & Gerardi, 2012; Parsons & Ressler, 2013). We were more interested in the clinical value of enhancement studies and focused on clinical questions such as: What are the proposed working mechanisms of different enhancers? What is known about enhancement effects of different pharmacological agents in PTSD patients? What would be the feasibility of these enhancers in clinical practice? Therefore, we focused on describing pharmacological agents that were studied in PTSD patients and administered in addition to (at least one) exposure-based treatment session, with the aim to enhance extinction or reconsolidation processes. This resulted in reviewing four different pharmacological enhancers: d-cycloserine, MDMA, hydrocortisone, and propranolol.

## d-cycloserine

### Proposed working mechanism of d-cycloserine

Fear extinction has been linked to *N*-methyl-d-aspartate (NMDA) glutamatergic receptor activity in the basolateral amygdala (Norberg, Krystal, & Tolin, 2008). Animal research suggested that NMDA receptor agonists, such as the partial agonist d-cycloserine (DCS), can enhance extinction effects (Richardson, Ledgerwood, & Cranney, 2004; Walker et al., 2002). Translating the positive findings in animals to humans, clinical studies in diverse anxiety-disordered clinical populations [e.g., in acrophobia (Ressler et al., 2004), social phobia (Hofmann et al., 2006) and panic disorder (Otto et al., 2010)] showed that fear extinction is indeed facilitated by supplementing exposure therapy with DCS (see for review: Bontempo, Panza, & Bloch, 2012; Norberg et al., 2008). The beneficial effects of DCS are attributable to extinction enhancement and not to anxiolytic effects of the drug, since both animal and human studies showed that fear expression (i.e., freezing in animals and subjective fear in humans) during extinction/exposure is not influenced by DCS (Kushner et al., 2007; Ressler et al., 2004; Walker et al., 2002).

### DCS enhancement of exposure-based treatment in PTSD patients

To date, two studies examined the additional effect on DCS in PE therapy for PTSD. De Kleine, Hendriks, Kusters, Broekman, and van Minnen (2012) investigated the effect of DCS in a mostly women, mixed-trauma civilian sample. Sixty-seven participants were randomized to receive either DCS (50 mg, N=34, see Table 1) or identical looking placebo (N=33) 1 hour prior to each imaginal exposure session (max. nine enhanced sessions). Exposure therapy was delivered in adherence to the PE manual (Foa & Rothbaum, 1998), and this included imaginal exposure therapy sessions that were enhanced with DCS. Overall, no enhancement effects of DCS were found: irrespective of treatment condition, symptoms declined over time. Clinician Administered PTSD Scale (CAPS) scores dropped on average with 28 (DCS) and 20 (Placebo) points from pre- to posttreatment. Looking at treatment response, an effect in favor of DCS was found. In the intent-to-treat sample, 64% in the DCS group showed response [defined as a minimum of 10 points decrease on CAPS scores (Schnurr et al., 2007)], compared to 38% in the placebo group. In the completers group, these numbers were overall higher, but still favored DCS (88% versus 62%). What is more, de Kleine et al. found that a large proportion of patients (approximately 40%) could be considered early completers, i.e., those patients could end treatment before the eighth session because of remission. When comparing these early completers to those that needed all treatment sessions (regular completers), an interesting difference emerged. For the early completers, no difference between those who received DCS and those who received placebo was found. In contrast, for the regular completers, those who received DCS showed better treatment outcome (30 point CAPS decline) than those who received placebo (6 point CAPS decline). The two subgroups (early vs. regular completers) did not differ on baseline characteristics as trauma type or comorbidity, but regular completers had more PTSD symptoms on baseline compared to early completers. Based on these findings, the authors tentatively concluded that DCS seems promising for severe PTSD patients who do not initially respond to exposure therapy.

**Table 1:** Characteristics and outcome of reviewed enhancement studies

Author (year)	Cognitive enhancer	Population	Study design	Intervention	Outcome (CAPS)	Other outcome measure	Comments
De Kleine et al. 2012	DCS	Civilian, 79% women, mixed trauma, N=67	RCT; 50 mg DCS or placebo 60 minutes prior to (max) 9 prolonged exposure sessions	Prolonged exposure; imaginal exposure (30–45 minutes) & exposure in vivo; homework assignments; 1 psycho-education and 9 exposure sessions.	No significant time×group effects. *Pretreatment:* DCS: 61.8 Placebo: 73.8 *Posttreatment* (intent-to-treat; model means): DCS: 34.3 Placebo: 53.7	Self-report (PSS-SR): no time×group differences.	
Litz et al. 2012	DCS	Veteran, male, N=26	RCT; 50 mg DCS or placebo 30 minutes prior to 4 exposure sessions	Brief exposure therapy; Imaginal exposure (50 minutes); 1 psycho-education, 4 exposure and 1 relapse prevention session.	Significant time×group effect, in favor of placebo. *Pretreatment:* DCS: 69.9 Placebo: 73.4 *Posttreatment* (intent-to-treat): DCS: 72.3 Placebo: 53.7	Self-report (PCL): significant time×group effect, in favor of placebo.	
Bouso, Doblin, Farre, Alcazar, & Gomez-Jarabo, 2008	MDMA	Civilian, women, sexual assault, N=6	RCT; 50 mg MDMA, 75 mg MDMA or placebo prior to 1 experimental session	Confrontation with the traumatic event, discussion of narrative and new insights, experience-based (6 hours); 1 session			No results available
Mithoefer et al. 2011	MDMA	Civilian, 85% women, mixed trauma, N=20	RCT; 125 mg (+62.5 mg) MDMA or placebo prior to 2 exposure-based sessions	Relaxation, experience-based, introspection and discussion of experiences (8–10 hours); 2 introductory sessions, 2 MDMA/placebo enhanced sessions, 4 integration sessions after each enhanced session (8 in total).*	Significant time×group effect, in favor of MDMA. *Pretreatment:* MDMA: 79.2 Placebo: 79.6 *Posttreatment:* MDMA: 29.3 Placebo: 66.8	Self-report (IES-R): Significant time×group effect, in favor of MDMA.	Results were maintained at follow-up (Mithoefer et al., 2013)
Oehen et al. 2013	MDMA	Civilian, 83% women, mixed trauma, N=12	RCT; 125 mg+62.5 mg MDMA or active placebo 25+12.5 mg MDMA prior to 3 exposure-based sessions	Relaxation, experience- based, introspection and discussion of experiences (8–10 hours); 2 introductory sessions; 3 MDMA/active placebo enhanced sessions, 3 integration sessions after each enhanced session (9 in total).	No significant time×group effect. *Pretreatment:* MDMA: 66.4 Placebo: 63.4 *Posttreatment:* MDMA: 50.8 Placebo: 66.5	Self-report (IES-R): significant time×group effect, in favor of MDMA.	
Brunet et al. 2008	Propranolol	Civilian, 52% women, mixed trauma, N=19	RCT; 40 mg short-acting+60 mg long-acting propranolol; immediately after 1 traumatic reactivation session	Traumatic memory reactivation; written description of index trauma (20 minutes); 1 session.	N/A	Physiological outcome: lower heart rate and skin conductance in response to trauma script in propranolol group.	
Yehuda et al. 2010	Hydrocortisone	Veteran, male, N=2	Controlled case study; 30 mg hydrocortisone or placebo 30 minutes prior to 8 prolonged exposure sessions	Prolonged exposure; imaginal exposure (60 minutes) & exposure in vivo; homework assignments; 2 psycho-education and 8 exposure sessions.	*Pretreatment:* Hydrocortisone: 97.0 Placebo: 94.0 *Posttreatment:* Hydrocortisone: 43.0 Placebo: 52.0	Self-report (PSS-SR): more symptom decline in hydrocortisone treated than placebo treated patient.	
Suris et al. 2010	Hydrocortisone	Veteran, male, N=20	RCT; 4mg/kg hydrocortisone or placebo immediately after 1 memory reactivation session	Traumatic memory reactivation; written account of 2 “worst” traumatic memories; 1 session.	N/A	Self-report (IES-R): lower avoidance/numbing symptoms in the hydrocortisone group compared to placebo.	
Brunet et al. 2011	Propranolol	Civilian, 68% women, mixed trauma, N=28	Open label; 0.67 mg/kg short-acting+1 mg/kg long-acting propranolol (modal dose resp. 40 and 60 mg), 90 minutes prior to 6 sessions	Traumatic memory reactivation; reading aloud a written account of the index trauma (<15–20 minutes); 6 sessions.	*Pretreatment*: Propranolol: 71.8 *Posttreatment*: Propranolol: 45.8	Self-report (PCL): decline of self-reported PTSD symptoms.	
	Propranolol	Civilian, 71% women, mixed trauma, N=7	Open label; 40 mg short-acting+80 mg long-acting (LA) propranolol, 90 minutes prior to 6 sessions	Traumatic memory reactivation; renarrating an oral account of the index trauma (<15–20 minutes); 6 sessions.	*Pretreatment*: Propranolol: 68.4 *Posttreatment*: Propranolol: 35.6		
	Propranolol	Civilian, 71% women, disaster survivors, N=7 (treated)	Open label; 40 mg short-acting+80 mg LA propranolol 90 minutes prior to session 1, 80 mg LA propranolol 90 minutes prior to sessions 2–6	Traumatic memory reactivation; reading aloud a written account of the index trauma (<15–20 minutes); 6 sessions.	N/A	Self-report (PCL): more symptom decline in treated group than non-treated group.	

* Additional integration sessions were permitted if needed. Seven participants in the MDMA group received additional sessions (20 sessions in total), compared to 1 participant (1 session).

DCS: d-cycloserine; MDMA: ±3,4-methylenedioxymethamphetamine; RCT: Randomized clinical trial; CAPS: Clinician Administered PTSD Scale; N/A: not applicable; PSS-SR: posttraumatic Stress-Scale Self-Report; PCL: PTSD checklist; IES-R: Impact of event scale—revised form.

However, a second study on DCS enhancement of exposure therapy in PTSD patients showed different outcome. Litz and colleagues (2012) examined the beneficial effects of DCS in a male, veteran population. Twenty-six participants were randomized to receive either DCS (50 mg; N=13) or placebo (N=13) 30 minutes prior to four imaginal exposure sessions. Litz and colleagues found that the placebo group had better outcome than the DCS group on both self-reported and clinician assessed PTSD symptoms. On average, CAPS scores declined 20 points in the placebo condition, but increased with 2 points in the DSC condition. For treatment response (>10 points decrease on CAPS scores; Schnurr et al., 2007), a similar pattern was found, 70% response in the placebo condition and only 30% in the DCS condition.

### Dosing, administration, adverse effects and contra-indications for DCS enhancement

DCS was originally approved by the U.S. Food and Drug Administration as an antibiotic for the treatment of tuberculosis. Generally, it is dosed at 500–1,000 mg twice daily. Peak blood levels occur within 4–8 hours after oral dosing, with a half-life time of 10 hours (Hardman & Limbird, 2001). As an enhancer to exposure therapy, DCS is single dosed before exposure sessions in low doses (in both PTSD trials 50 mg). Dosage, dose timing or number of doses have varied and were not significantly related to outcome in DCS enhancement trials across anxiety disorders (Bontempo et al., 2012).

DCS is orally taken and usually well tolerated. There is no need to physically monitor patients after DCS intake. Adverse reactions seem to be related to higher dosages of the drug (i.e., more than 500 mg daily). Side effects that have been observed in these high dosages involve nervous system symptoms (e.g., convulsions, drowsiness, headache, tremor), cardiovascular problems, allergy, and skin rash. However, no serious adverse event was reported in the PTSD trials or in any other clinical trial with DCS as enhancer of exposure therapy (and thus with much lower dosages).

Looking at contra-indications for DCS enhancement, patients with (a history of) epileptic seizures or concurrent alcohol dependence or abuse were excluded from participation in the PTSD trials, because of the risk of epileptic episodes with DCS administration (again, especially in higher doses), which is increased with alcohol. Patients who used antidepressants were not excluded in both PTSD trials, but they were required to be on a stable dose prior to enrollment. In lab-rats it was found that DCS did not facilitate extinction learning in rats previously exposed to the tricyclic antidepressant imipramine (Werner-Seidler & Richardson, 2007), but no evidence was found for negative interaction effects between antidepressant medication and DCS enhancement effects in patients with PTSD (de Kleine et al., 2012) or other anxiety disorders (Hofmann et al., 2006; Kushner et al., 2007; Storch et al., 2007).

### Discussion

Granted that DCS was found to enhance exposure therapy across anxiety disorders (Bontempo et al., 2012; Norberg et al., 2008), so far findings in PTSD populations are inconclusive. It is difficult to combine the findings of the De Kleine et al. and Litz et al. studies, because the studies differed largely with respect to population (e.g., civilian vs. veteran) and methodology (e.g., different lengths of treatment protocol and varying times of DCS administration). In line with the findings of de Kleine and colleagues (2012), there is some evidence that DCS is especially beneficial in severely disordered patients (Guastella, Dadds, Lovibond, Mitchell, & Richardson, 2007; Siegmund et al., 2011).

The proposition that DCS enhances consolidation of extinction learning suggests that in case of within session extinction (i.e., good extinction) DCS has beneficial effects, but also that in absence of within session extinction (i.e., no extinction), DCS might have undesirable effects by consolidation of the fear memory. Indeed, Smits and colleagues (2013) found in an acrophobic sample that DCS was superior to placebo in sessions wherein within session extinction occurred but lead to detrimental effects in sessions without extinction. Likewise, in PTSD patients, Litz et al. (2012) found indicators that those who received DCS showed less within session extinction compared to those who received placebo, and Litz and colleagues suggest that this may be underlying their finding that placebo outperformed DCS.

This raises the questions whether DCS could not be better administered postsession and only when good extinction learning took place. In animal studies, it was found that postextinction training administration of DCS could also enhance extinction effects (Ledgerwood, Richardson, & Cranney, 2003). But, the first trials in humans failed to find beneficial effects of postsession administered DCS (Tart et al., 2013), except in those patients who showed good within session habituation (Smits et al., 2013). And, despite the apparent logic of administering DCS only after successful sessions, research on the predictive value of within session extinction for overall treatment outcome is indecisive (Craske et al., 2008; van Minnen & Hagenaars, 2002), and the question what exactly defines a successful exposure session has yet to be answered.

Reflecting on the clinical utility of DCS, it seems a good candidate to implement in routine clinical care. All in all, DCS seems safe, usually well tolerated and easy to administer in routine clinical care settings. There are no major drug specific contra-indications, although, given the high incidence of alcohol abuse in PTSD populations (Mills, Teesson, Ross, & Peters, 2006), extra attention should be given to alcohol use during DCS enhanced exposure therapy.

## MDMA

### Proposed working mechanism of MDMA enhancement

MDMA (±3,4-methylenedioxymethamphetamine) is a substituted phenetylamine and binds and reverses monoamine transporters, resulting in serotonin release and activation of the 5-HT receptor, and to a lesser extent to the release of norepinephrine and dopamine (Johansen & Krebs, 2009; Sessa, 2008). MDMA is known in the public domain as the recreational drug ecstasy. The drug has profound subjective effects as feelings of euphoria and well-being, heightened senses, and closeness to others (Cami et al., 2000; Kolbrich et al., 2008). Johansen and Krebs (2009) suggested that MDMA could enhance extinction learning via three different mechanisms. First, MDMA might enhance extinction learning via increased activity in the ventromedial prefrontal cortical (vmPFC) and decreased amygdala activity; two interconnected brain regions that have been found to be critical for extinction learning (Phelps, Delgado, Nearing, & LeDoux, 2004). Second, MDMA might enhance extinction learning via enhanced cortisol and norepinephrine levels. And a third, more indirect way, may be that MDMA increases oxytocin levels, which may strengthen the therapeutic alliance, and thereby facilitate extinction learning.

To date, no experimental study examined whether MDMA could enhance extinction learning. There is circumstantial evidence for the hypothesis that MDMA strengthens the therapeutic alliance via enhanced oxytocin release. In both animal (Thompson, Callaghan, Hunt, Cornish, & McGregor, 2007) and human studies (Dumont et al., 2009; Hysek, Domes, & Liechti, 2012) it was found that MDMA leads to higher blood oxytocin levels, and in humans it was shown that blood oxytocin levels were strongly and positively correlated to subjective pro-social feelings.

### MDMA enhancement of exposure-based treatment in PTSD patients

Three studies examined the augmentation effects of MDMA of exposure-based treatment in PTSD patients. Bouso and colleagues (2008) planned to examine the beneficial effects of MDMA paired with psychological treatment in 29 patients with chronic PTSD following sexual assault, who had failed to respond to previous treatment. But due to political pressure, they were forced to close their double-blind randomized placebo-controlled trial after including only six participants and no results are available.

In the first completed study, Mithoefer and colleagues (2011) examined MDMA assisted treatment in 20 PTSD patients. All patients had not responded to previous psychotherapy or medication. Participants were randomized to receive either MDMA (125 mg, with the possibility of a supplemental dose of 62.5 mg; N=12) or pill placebo (N=8) adjunctive to two 8–10 hour individual psychotherapy sessions. Prior to the enhanced session, participants engaged in two sessions aimed at establishing a therapeutic alliance. As described in the treatment manual (available from www.maps.org), the enhanced sessions had a non-directive character: participants were instructed to close their eyes, relax (with the help of music) and allow the inner experience to unfold. Exposure to the traumatic experience occurred spontaneously. After each MDMA enhanced session, four integration sessions took place focusing on discussion of the experiences in the experimental sessions and further emotional processing of the traumatic experience. MDMA enhanced therapy outperformed placebo enhanced therapy on both clinician rated and self-reported PTSD symptoms. On average, CAPS scores in the MDMA group decreased 50 points from pre- to posttreatment, compared to 13 points in the placebo group. Further, over 80% of participants in the MDMA group (10 out of 12 patients) lost their PTSD diagnosis, compared to 25% (two out of eight patients) in the placebo group. The double-blind phase of the study was followed up by a cross-over open-label phase, so that in the end 19 participants received two or three MDMA enhanced treatment sessions. Long-term follow-up on 16 participants showed that for 14 of them, benefits lasted over time (Mithoefer et al., 2013). All in all, these appeared to be very promising findings. However, some important methodological limitations require noting. First, due to the profound effects of MDMA compared to pill placebo, it was impossible to keep participants and therapists blind to treatment condition. Second, participants in the MDMA group received more supplementary (exposure) sessions after the experimental sessions than participants in the placebo group. Third, the psychotherapy component was not an empirically supported treatment delivered in a standard manner.

In the most recent pilot study (Oehen, Traber, Widmer, & Schnyder, 2013), 12 treatment refractory PTSD patients were randomly allocated to receive either MDMA (125 and 62.5 mg 2.5 hours later; N=8) or active placebo (25 and 12.5 mg 2.5 hours later; N=4) supplementary to three 8-hour individual psychotherapy sessions. Oehen et al. used roughly the same study protocol as Mithoefer and colleagues and exactly the same treatment protocol. However, the results of this study were less profound in favoring MDMA over placebo. There was a significant interaction of group by time for self-reported PTSD symptoms in favor of MDMA, and a trend towards better outcome for the MDMA group in clinician rated scores (*p=*0.066). But, compared to the Mithoefer study, change in CAPS scores from baseline to posttreatment was small (16 points decrease in the MDMA group and 3 points increase in the placebo group), and, notably, all participants still fulfilled PTSD diagnostic criteria at posttreatment.

### Dosing, administration, adverse effects and contra-indications for MDMA enhancement

A typical (recreational or therapeutic) dose of MDMA is 125 mg, and was also used in the PTSD studies. It is orally administered and comes in the form of tablets, capsules or powder. MDMA is detectable in the blood within 30 minutes after intake, peak blood levels are 1–2 hours after intake and MDMA has a half-life of about 6–8 hours (Green, Mechan, Elliott, O'Shea, & Colado, 2003).

Acute adverse effects are commonly reported, including jaw clenching, grinding of the teeth, nausea, tremor and feelings of tension and anxiety (Kolbrich et al., 2008). After MDMA usage, there is a period of neurochemical recovery characterized by anhedonia, lethargy and depression. Acute adverse effects were indeed reported in the PTSD trials, but there were no serious adverse events. Further, neurotoxicity has been demonstrated in animals, and suggested in humans, but functional impairment in humans has not been convincingly related to MDMA (Dumont & Verkes, 2006). Importantly, not all neuropharmocological actions of MDMA are well comprehended (Green, Marsden, & Fone, 2008).

The cardiovascular effects of the drug are important to note. MDMA leads to higher blood pressure and elevated heart rates (Dumont & Verkes, 2006). Thus, MDMA is unsuitable for PTSD patients with cardiovascular problems, requires physical examination before administration (stress-electrocardiogram), and physical monitoring and the presence of a physician during MDMA enhanced treatment sessions. Further, in the PTSD trials, participants were required to taper all psychotropic medication prior to enrollment. Interestingly, empirical data suggests that medication that inhibits serotonin (5-HT) and norepinephrine (NE) uptake (respectively SSRI's and SNRI's), drugs commonly used by PTSD patients, attenuate the effects of MDMA (Hysek et al., 2012; Liechti, Baumann, Gamma, & Vollenweider, 2000).

### Discussion

In conclusion, even though the initial findings of MDMA enhancement appear promising, more controlled studies on MDMA enhanced treatment are necessary to draw reliable conclusions on the efficacy of this combined treatment strategy. In the studies so far, MDMA was not paired to a proven effective treatment strategy, i.e., prolonged exposure (Foa & Rothbaum, 1998). Critically reflecting on the treatment manual used in MDMA trials, it is questionable whether the non-directive, explorative intervention should be considered exposure, as it diverges from evidence-based exposure treatments such as PE. Preferably, new trials would combine MDMA with those established exposure treatments.

With respect to one of the proposed working mechanisms of MDMA enhancement, that it enhances extinction learning indirectly via improvement of the therapeutic alliance due to heightened oxytocin levels, the question arises whether direct administration of oxytocin would also enhance exposure therapy. Olff and colleagues (2010) suggest that it might, and may even be more powerful than MDMA. Currently, Olff's group is examining oxytocin enhancement of exposure therapy in refugees suffering from PTSD.

Reflecting on the feasibility of MDMA enhancement, all together there are questions regarding safety and tolerability. Considering the high rate of comorbid depression in PTSD patients (Kessler et al., 2005), the negative mood effects post-MDMA intake calls for caution. Further, a substantial proportion of PTSD patients use SSRI's or SNRI's which appear to be incompatible with MDMA enhancement. Implementation in routine clinical care is further complicated by the need for physical monitoring during MDMA enhancement, especially with its known cardiac effects. And notably, the use of MDMA as an adjunctive to treatment is not without controversy, as its use as a recreational drug (“ecstasy”) is criminalized in most countries. The alternative of oxytocin enhancement may have better clinical utility, considering that it is easily administered e.g., as a nasal spray and produces little adverse effects (MacDonald et al., 2011).

## Hydrocortisone

### Proposed working mechanism

Hydrocortisone is a synthetic glucocorticoide that mimics the effects of cortisol. In situations of stress, the hypothalamus–pituitary–adrenal (HPA) axis is activated, which results in the release of glucocorticoide hormones, i.e., cortisol. It is well-established that cortisol has (complex) effects on learning and memory (see for a review on glucocorticoide: de Quervain, Aerni, Schelling, & Roozendaal, 2009). First, glucocorticoids play a role in the consolidation of extinction learning. It has been shown that administration of glucorticoids facilitates consolidation of extinction learning, while suppression of glucorticoid function impairs extinction learning. Second, glucorticoids impair the retrieval of emotional memory. In PTSD patients it was found that low-dose cortisol administration as stand-alone treatment resulted in reduction of re-experiencing symptoms, i.e., unwanted retrieval of emotional memories (Aerni et al., 2004). Enhancement of exposure therapy with hydrocortisone could be beneficial via both mechanisms: (1) enhanced consolidation of extinction learning, and (2) inhibition of emotional memory retrieval. The latter may reduce distress after exposure sessions, because retrieval of the emotionally disturbing traumatic memory that was targeted during an exposure session is inhibited (Yehuda, Bierer, Pratchett, & Malowney, 2010).

Two randomized studies provided support for exposure augmentation with hydrocortisone in anxiety-disordered patients. Hydrocortisone given prior to exposure sessions proved to augment treatment effects in patients suffering from spider phobia (Soravia et al., 2006) and acrophobia (de Quervain et al., 2011).

### Hydrocortisone enhancement of exposure-based treatment in PTSD patients

To date, there are no randomized clinical trials on the augmentation effects of exposure therapy with hydrocortisone in PTSD patients. Two large scaled clinical studies by Yehuda and colleagues are currently including participants (Clinicaltrials.gov identifiers NCT01525680 and NCT01090518). Grounding their upcoming trials, Yehuda and colleagues (2010) published a case study on hydrocortisone enhancement in two male veterans suffering from severe PTSD and comorbid depressive disorder. Participants were treated with PE therapy and either hydrocortisone (30 mg, oral, 30 minutes prior to exposure session 3–10) or placebo (following the same time schedule). The patient who received hydrocortisone improved more over the course of treatment than the patient who received placebo (54 versus 40 points reduction on CAPS), specifically with respect to avoidance symptoms.

In an experimental design, Suris and colleagues (2010) investigated the effects of hydrocortisone in 20 male veterans suffering from PTSD. Participants received either hydrocortisone (intravenous, 4 mg/kg) or placebo, directly after one exposure-based session. During this session, participants wrote a description of their two most traumatic events and identified from a list bodily sensations they experienced during these events. Afterwards, a member of the research team composed a 30-second script portraying each event. A week later, scripts were presented to participants while physiological responses (heart rate, skin conductance and corrugator and frontalis muscle electromyogram) were measured. Subsequently, they filled out questionnaires on PTSD and depressive symptoms. Suris et al. found that, compared to placebo, participants who received hydrocortisone had lower self-reported PTSD avoidance and numbing symptoms at script presentation. There were no significant differences on any other PTSD symptom cluster or on the physiological measures. Also, at the 1 month follow-up assessment, no group differences on any outcome were found. In the discussion section of their article, the authors list several limitations of their study. Besides the low power, they critically reflect on their study design, and suggest that with a higher dose and more enhanced treatment sessions effects may have been larger and more lasting.

### Dosing, administration, adverse effects and contra-indications for hydrocortisone enhancement

Hydrocortisone can be administered orally, intramuscularly or intravenously. It is used in the treatment of inflammation disease, e.g., severe allergies, arthritis or asthma, and as a replacement strategy in the chronic endocrine disorder Addison's disease. Therapeutic doses vary between 20 and 500 mg daily, depending on the specific disease treated. Hydrocortisone is well absorbed after oral administration, achieving peak blood concentrations after 1 hour, with a half-life of approximately 1.5 hours. Dosing and administration of hydrocortisone given in supplementary fashion to exposure-based treatment in PTSD patients have varied largely [eight times, 60 minutes prior to exposure, orally 30 mg (Yehuda et al., 2010) versus one time, immediately after exposure, intravenously 4 mg/kg (Suris, North, Adinoff, Powell, & Greene, 2010)]. In the two non-PTSD exposure enhancement trials, hydrocortisone was given three or four times, 60 minutes prior to exposure, orally and dosed at 10 and 20 mg (de Quervain et al., 2011; Soravia et al., 2006).

Low doses of hydrocortisone (e.g., 10–30 mg once a week) do not cause major side effects, nor do they suppress endogenous cortisol levels (de Quervain et al., 2009). There is no need to physically monitor patients after hydrocortisone intake. Besides the regular exclusion criteria for drug trials (e.g., pregnancy, severe medical illness or drug-hypersensitivity) there appear to be no major drug specific exclusion criteria for hydrocortisone enhanced treatment relevant to the PTSD population. While participants who used psychotropic medication were excluded from non-PTSD trials (de Quervain et al., 2011; Soravia et al., 2006), in upcoming PTSD trials participants with (stable) psychotropic medication are included.

### Discussion

The evidence for beneficial effects of hydrocortisone enhancement of exposure therapy in PTSD patients is very limited. Efficacy studies in other anxiety disorders showed augmentation of exposure effects, but upcoming controlled trials will have to show whether this generalizes to PTSD patients.

In PTSD patients, it appears that hydrocortisone specifically affects avoidance symptoms. Avoidance has been implicated in the development and maintenance of PTSD (Foa, Hembree, & Rothbaum, 2007). Hypothetically, decline of avoidance symptoms over the course of treatment could improve compliance with exposure sessions and in that way hasten or improve beneficial treatment effects (Yehuda et al., 2010). Further, it is of interest to note that hydrocortisone may have acute anxiolytic effects. A clinical trial in patients with social phobia showed that hydrocortisone (25 mg) given 1 hour prior to an exposure task reduced self-reported fear (Soravia et al., 2006). What the effect is of presession hydrocortisone on fear levels during exposure therapy in PTSD patients, and if and how this influences treatment efficacy, has yet to be established. The proposition that hydrocortisone impairs retrieval of the traumatic memory targeted during an exposure session may reduce postexposure distress and enhance treatment acceptability. Given the high incidence of (early) exposure treatment dropout (Bisson & Andrew, 2007), improving treatment acceptability is of great relevance.

Reflecting on its clinical utility, hydrocortisone seems safe, well tolerated and easy to administer. There are no major drug specific exclusion criteria relevant to the PTSD population. So, albeit premature, hydrocortisone appears to be feasible to implement in routine clinical care.

## Propranolol

### Proposed working mechanism

Propranolol is a synthetic β-adrenergic receptor blocker that crosses the blood brain barrier and has both peripheral noradrenergic effects and central inhibitory effects on protein synthesis. Protein synthesis is necessary to (re)consolidate new memories and a protein synthesis inhibitor, such as propranolol, could interfere with this process (Davis & Squire, 1984). Indeed, experimental studies in non-clinical samples demonstrated that propranolol has effects on memory reconsolidation (see for overview: Lonergan, Olivera-Figueroa, Pitman, & Brunet, 2012). For instance, Kindt and colleagues (2009) found that disruption of reconsolidation by oral administration of propranolol resulted in diminished fear responses to conditioned stimuli. Even though there is growing interest in (disruption of) reconsolidation processes, clinical studies in anxiety-disordered patients are limited to a couple of studies in PTSD patients.

### Propranolol enhancement of exposure-based treatment in PTSD patients

In a randomized clinical trial, Brunet and colleagues (2008) examined the effects of propranolol given directly after one exposure-based session in 19 chronic PTSD patients following mixed trauma. Participants received either propranolol (40 mg short acting (SA) and 2 hours later 60 mg long acting (LA); N=9) or identical looking placebo (following the same time schedule; N=10) immediately after an exposure-based session. During this 20-minute session, participants described, in writing on a standard script preparation form, two events that caused their PTSD and provided details on request. Afterwards, a member of the research team composed and recorded a 30-second during script portraying each event. A week later, participants were exposed to the trauma scripts while physiological responses (heart rate, skin conductance and left corrugators electromyogram, i.e., facial frowning muscle) were measured. Participants who had received propranolol postsession responded with lower heart rate and skin conductance than participants who had received placebo. Unfortunately, information on long-term effects, i.e., PTSD symptoms was not included so there is no information regarding the maintenance of these effects and if there was any effect on PTSD symptoms.

There is support for the efficacy of propranolol as a treatment enhancement strategy from three open-label studies by Brunet and colleagues (2011). In the first study, 28 patients with chronic PTSD following mixed trauma received six propranolol enhanced exposure-based treatment sessions. Ninety minutes prior to the first session participants received 0.69 mg/kg SA propranolol, after 90 minutes they received a subsequent dose of 1 mg/kg LA propranolol. In this first session, they provided a written account of the traumatic event leading to their PTSD. In the subsequent enhanced treatment sessions, participants received both SA and LA propranolol 90 minutes prior to the start of the session and read aloud their traumatic account to an interviewer, as if the event was happening in the here and now. Sessions lasted approximately 15–20 minutes. At posttreatment, PTSD symptoms were significantly lower than at pretreatment (mean CAPS decline from pre- to posttreatment of 26 points) and 20 participants (71%) no longer met the criteria for PTSD. In the second open study, a similar protocol was followed, only now participants provided an oral instead of a written account of the index trauma and propranolol was given in fixed doses of 40 mg SA and 80 mg LA. Participants were seven chronic PTSD sufferers with mixed-trauma history. Again, there was a significant drop in PTSD symptoms (mean CAPS scores: 68 at baseline and 36 posttreatment). Comparable to the first study, 71% (five participants) no longer met PTSD diagnostic criteria. In study 3, 32 participants self-selected to receive propranolol enhanced treatment (N=7) or no treatment at all (N=25). Participants all suffered from PTSD following the September 2001 industrial disaster in Toulouse, France. The protocol was similar to study 1 with slight differences in propranolol dosing (see Table 1) and there was no administration of CAPS interviews. In line with the previous findings, for those participants who received propranolol enhanced treatment, self-reported PTSD symptoms declined over time. Six out of seven treated participants (86%) lost their PTSD diagnosis, compared to only two out of 25 (8%) in the control group.

### Dosing, administration, adverse effects and contra-indications for propranolol enhancement

Propranolol lowers heart rate and relaxes blood vessels to improve blood flow and decrease blood pressure. As such, it is used for treatment of hypertension, angina pectoris, migraine and tremor. In addition, propranolol has anxiolytic effects (it reduces physical anxiety symptoms such as trembling and heart pounding) and is used to reduce performance related anxiety. Generally, doses vary between 40 and 240 mg daily, depending on treatment condition. In PTSD trials, propranolol was single dosed directly before or after exposure-based treatment session(s) with doses varying between 80 and 120 mg. Propranolol is rapidly absorbed. For SA propranolol, peak blood levels occur within 1–2 hours after ingestion and half-life is approximately 3–6 hours, while for LA propranolol this is respectively 5 hours and between 10 and 20 hours.

Adverse reactions to propranolol appear to be dose related and include light headedness, weakness, fatigue, bradycardia, congestive heart failure, hypotension, nausea and vomiting. In PTSD patients, when given as a treatment enhancer, no serious adverse events were reported and side effects were restricted to mild sedation (Brunet et al., 2011).

Propranolol is orally administered. Because propranolol lowers blood pressure, in the Brunet trials blood pressure was monitored after the first propranolol intake. If systolic blood pressure levels did not drop below a certain point (100 mmHg), participants received the subsequent doses. Because of its cardiovascular effects, people with low blood pressure, a (family) history of cardiac problems (e.g., heart failure, heart block or certain cardiac arrhythmias) were excluded from participation in propranolol studies. Like propranolol, alcohol relaxes blood vessels, and simultaneous use may cause problems. In the open-label trials, participants who used medication that could involve dangerous interactions with propranolol were excluded from participation. Notably, this includes antidepressants that are cytochrome P450 2D6 inhibitors, such as the SSRI paroxetine.

### Discussion

Combined, the findings with propranolol enhancement are promising and we await a placebo-controlled randomized clinical trial on propranolol enhancement in PTSD patients. However, due to the uncontrolled methodology of the propranolol treatment enhancement studies, the question of whether the observed benefits were a consequence of propranolol enhancement or the psychological intervention in itself remains unanswered.

Likewise, regarding the proposed working mechanism, it is unclear from Brunet's studies whether propranolol blocked fear-memory reconsolidation or, alternatively, enhanced extinction consolidation. Parsons & Ressler (2013) question whether propranolol would succeed in reconsolidation blockage in PTSD patients, based on research findings that older memories are more difficult to influence after retrieval (Milekic & Alberini, 2002) and that repeated retrieval (i.e., re-experiences) can strengthen fear memories and make them more resistant to extinction and reconsolidation effects (Suzuki et al., 2004). The upcoming controlled trial of Brunet's group (N=50, six enhanced sessions with 40 mg SA/60 mg LA propranolol, Clinicaltrials.gov identifier NCT01127568) will provide us with more information on the potential of propranolol as a treatment enhancer in PTSD patients¯

Considering the feasibility of propranolol for exposure treatment enhancement in PTSD patients, propranolol appears to be well tolerated and easy to administer. However, its cardiovascular effects require caution in patients with (a family history) of cardiovascular problems, and alcohol use should be carefully monitored during propranolol enhanced treatment, especially considering that alcohol abuse is a common problem in PTSD patients (Mills et al., 2006). PTSD patients who use SSRI's have been excluded from propranolol enhancement studies (and will be excluded in upcoming trials, see www.clinicaltrials.gov), because of potential dangerous drug-drug interactions. This may limit generalizability, considering that in routine clinical care many PTSD patients receive psychotropic medication, including SSRI's, prior to the start of psychological treatment. For instance, in a large randomized clinical trial, over 75% of PTSD patients were receiving psychotropic medication at baseline (Schnurr et al., 2007).

## General discussion

In this article, we reviewed the clinical data on treatment enhancement of exposure-based therapies in PTSD patients. Enhancement of learning processes during exposure therapy is an emerging research field and shows promise in improving treatment efficacy for PTSD. Reviewing the literature to date also revealed some challenges for future research, discussed below.

Focusing on those enhancers that were given in addition to exposure-based treatment sessions in patients suffering from PTSD, resulted in the review of four different pharmacological enhancers. The early stage of exposure treatment enhancement studies is reflected in the reviewed studies, which could largely be considered pilot work. Only a few studies were randomized clinical trials with adequate blinding and sample size [DCS: De Kleine et al. (2012); Litz et al. (2012); MDMA: Oehen et al. (2013)]. All studies differed from one another in greater or lesser extent, so that, to date, no study has been fully replicated. Further, the studies on MDMA were supported by one association (MAPS, multidisciplinary association of psychedelic studies) and the same research group conducted all the studies on propranolol enhancement. For most enhancers the empirical evidence is still limited and conclusions on efficacy are premature. Similarly, a conclusion about which enhancer has the most potential to enhance exposure therapy effects in PTSD patients cannot be drawn. To date, DCS has been studied most and proven efficacious across anxiety disorders, but results in PTSD studies were mixed, while for the other enhancers efficacy studies are still scarce.

Even though all reviewed studies paired the pharmacological agent to an exposure-based treatment session, studies varied widely in the nature and amount of exposure, and only two studies paired the agent to a full protocol of proven effective treatment strategy. De Kleine and colleagues (2012) paired DCS with PE therapy (Foa & Rothbaum, 1998), and Yehuda and colleagues (2010) added hydrocortisone to PE therapy. This variation in exposure dose is partly due to differences in proposed working mechanisms, extinction consolidation enhancement and/or reconsolidation blockage, which suggests respectively longer and shorter duration of trauma memory exposure. Note however, that even though extinction and reconsolidation are considered to be two distinct processes, there is also some evidence of overlapping properties (Parsons & Ressler, 2013), and it is not yet perfectly understood how both processes contribute to exposure therapy effects (Kindt & Soeter, 2013). We would like to encourage future enhancement trials to pair the cognitive enhancer with an empirically based treatment such as PE therapy. Only then it can be established whether augmentation with cognitive enhancers is superior to already proven effective treatment strategies. In this line, we suggest that authors provide a detailed description of the psychological intervention in their enhancement studies reports, so that readers may be able to understand exactly what was administered and judge its quality and propriety.

Almost all reviewed studies reported outcome on the CAPS scores, which allowed us to compare studies on symptom decline over time. In some studies, the decline of CAPS scores was spectacular (≥50 points; Mithoefer, Wagner, Mithoefer, Jerome, & Doblin, 2011; Yehuda et al., 2010), and well exceeded those of non-enhanced exposure trials, but note that these enhancement studies were less well controlled. In addition to reporting overall CAPS scores, we would like to argue that future enhancement studies report scores for PTSD symptom clusters (re-experiencing, hyperarousal and avoidance symptoms) separately. The two reviewed studies on hydrocortisone, showed that hydrocortisone appears to be beneficial primarily via diminishment of avoidance behavior. It would be interesting to learn whether enhancers differ in their effects on different symptom clusters. This would provide us with more information on possible mechanisms of action. Thinking about future directions for this field, it would be interesting to explore for which patients augmentation with a cognitive enhancer might be especially beneficial. Studies addressing individual differences (for instance: dissociative subtype of PTSD (American Psychiatric Association, 2013), comorbidity, trauma type, personality features) may enhance our understanding of treatment efficacy and ultimately contribute to treatment matching strategies.

Reflecting on the clinical utility of the cognitive enhancers, with the exception of MDMA, they seem to be safe and well tolerated. When used as a cognitive enhancer, the doses of DCS, hydrocortisone, and propranolol are relatively low and infrequent, while adverse effects of these pharmacological agents have been related to higher doses and frequent use. In this respect, they show advantage over exposure enhancement with SSRI's (Schneier et al., 2012), with which adverse effects are commonly reported. Given the high incidence of alcohol abuse in PTSD patients (Mills et al., 2006), close monitoring of alcohol intake during enhanced exposure treatment is advised. Although alcohol abuse is not considered to be a contra-indication for exposure treatment in PTSD patients (Mills et al., 2012; van Minnen, Harned, Zoellner, & Mills, 2012) the potential hazardous drug-drug interactions call for caution. Additionally, the use of concurrent psychotropic medication, specifically SSRI's, deserves extra attention in the light of exposure treatment enhancement. Considering that a large proportion of PTSD patients receive psychotropic medication, and that use of antidepressants (SSRI's) is an exclusion criterion for enhancement studies with propranolol and MDMA, generalizability of findings with these cognitive enhancers, and ultimately clinical utility, are limited. Last, there is some data from DCS enhancement showing that augmentation can also have detrimental effects when sessions lack extinction. For safe use in clinical care, it is important to gather more information on potential undesirable outcomes. Therefore, we urge authors to report symptom worsening in participants as adverse effects and researchers to further investigate process variables that affect outcome.

In the coming years the amount of studies in this exciting new field will expand quickly. New enhancers (yohimbine, oxytocin, methylene blue) will be studied, and previously studied enhancers will be examined in larger trials. A search in trial registries (www.clinicaltrials.gov; www.clinicaltrialsregister.eu) reveals that large(r) clinical trials on DCS (N>100), hydrocortisone (N=60), and propranolol (N=50) enhancement in PTSD patients are currently enrolling patients. Hopefully, translational designs will additionally provide us with more information on the mechanisms of action of exposure therapy (extinction and reconsolidation) and the possibility to enhance these processes with diverse pharmacological agents.

## References

[CIT0001] Aerni A, Traber R, Hock C, Roozendaal B, Schelling G, Papassotiropoulos A (2004). Low-dose cortisol for symptoms of posttraumatic stress disorder. American Journal of Psychiatry.

[CIT0002] American Psychiatric Association (2013). Diagnostic and statistical manual of mental disorders.

[CIT0003] Arntz A, Tiesema M, Kindt M (2007). Treatment of PTSD: A comparison of imaginal exposure with and without imagery rescripting. Journal of Behavior Therapy and Experimental Psychiatry.

[CIT0004] Bisson J, Andrew M (2007). Psychological treatment of post-traumatic stress disorder (PTSD). Cochrane Database of Systematic Reviews.

[CIT0005] Bontempo A, Panza K. E, Bloch M. H (2012). d-cycloserine augmentation of behavioral therapy for the treatment of anxiety disorders: A meta-analysis. Journal of Clinical Psychiatry.

[CIT0006] Bouso J. C, Doblin R, Farre M, Alcazar M. A, Gomez-Jarabo G (2008). MDMA-assisted psychotherapy using low doses in a small sample of women with chronic posttraumatic stress disorder. Journal of Psychoactive Drugs.

[CIT0007] Bouton M. E (1993). Context, time, and memory retrieval in the interference paradigms of Pavlovian learning. Psychological Bulletin.

[CIT0008] Bradley R, Greene J, Russ E, Dutra L, Westen D (2005). A multidimensional meta-analysis of psychotherapy for PTSD. American Journal of Psychiatry.

[CIT0009] Brunet A, Orr S. P, Tremblay J, Robertson K, Nader K, Pitman R. K (2008). Effect of post-retrieval propranolol on psychophysiologic responding during subsequent script-driven traumatic imagery in post-traumatic stress disorder. Journal of Psychiatric Research.

[CIT0010] Brunet A, Poundja J, Tremblay J, Bui E, Thomas E, Orr S. P (2011). Trauma reactivation under the influence of propranolol decreases posttraumatic stress symptoms and disorder: 3 open-label trials. Journal of Clinical Psychopharmacology.

[CIT0011] Cami J, Farre M, Mas M, Roset P. N, Poudevida S, Mas A (2000). Human pharmacology of 3,4-methylenedioxymethamphetamine (“ecstasy”): Psychomotor performance and subjective effects. Journal of Clinical Psychopharmacology.

[CIT0012] Choi D. C, Rothbaum B. O, Gerardi M, Ressler K. J (2010). Pharmacological enhancement of behavioral therapy: Focus on posttraumatic stress disorder. Current Topics in Behavioral Neurosciences.

[CIT0013] Craske M. G, Kircanski K, Zelikowsky M, Mystkowski J, Chowdhury N, Baker A (2008). Optimizing inhibitory learning during exposure therapy. Behaviour Research and Therapy.

[CIT0014] Davidson J, Pearlstein T, Londborg P, Brady K. T, Rothbaum B, Bell J (2001). Efficacy of sertraline in preventing relapse of posttraumatic stress disorder: Results of a 28-week double-blind, placebo-controlled study. American Journal of Psychiatry.

[CIT0015] Davis H. P, Squire L. R (1984). Protein synthesis and memory: A review. Psychological Bulletin.

[CIT0016] de Kleine R. A, Hendriks G.-J, Kusters W. J. C, Broekman T. G, van Minnen A (2012). A randomized placebo-controlled trial of d-cycloserine to enhance exposure therapy for posttraumatic stress disorder. Biological Psychiatry.

[CIT0017] de Quervain D. J, Aerni A, Schelling G, Roozendaal B (2009). Glucocorticoids and the regulation of memory in health and disease. Frontiers in Neuroendocrinology.

[CIT0018] de Quervain D. J, Bentz D, Michael T, Bolt O. C, Wiederhold B. K, Margraf J (2011). Glucocorticoids enhance extinction-based psychotherapy. Proceedings of the National Academy of Sciences of the United States of America.

[CIT0019] Debiec J, Ledoux J. E (2004). Disruption of reconsolidation but not consolidation of auditory fear conditioning by noradrenergic blockade in the amygdala. Neuroscience.

[CIT0020] Dumont G. J, Sweep F. C, van der Steen R, Hermsen R, Donders A. R, Touw D. J (2009). Increased oxytocin concentrations and prosocial feelings in humans after ecstasy (3,4-methylenedioxymethamphetamine) administration. Social Neuroscience.

[CIT0021] Dumont G. J, Verkes R. J (2006). A review of acute effects of 3,4-methylenedioxymethamphetamine in healthy volunteers. Journal of Psychopharmacology.

[CIT0022] Dunlop B. W, Mansson E, Gerardi M (2012). Pharmacological innovations for posttraumatic stress disorder and medication-enhanced psychotherapy. Current Pharmaceutical Design.

[CIT0023] Farach F. J, Pruitt L. D, Jun J. J, Jerud A. B, Zoellner L. A, Roy-Byrne P. P (2012). Pharmacological treatment of anxiety disorders: Current treatments and future directions. Journal of Anxiety Disorders.

[CIT0024] Foa E. B, Hembree E. A, Cahill S. P, Rauch S. A. M, Riggs D. S, Feeny N. C (2005). Randomized trial of prolonged exposure for posttraumatic stress disorder with and without cognitive restructuring: Outcome at academic and community clinics. Journal of Consulting and Clinical Psychology.

[CIT0025] Foa E. B, Hembree E. A, Rothbaum B. O (2007). Prolonged exposure therapy for PTSD: Emotional processing of traumatic experiences: Therapist guide.

[CIT0026] Foa E. B, Rothbaum B. O (1998). Treating the trauma of rape: Cognitive-behavioral therapy for PTSD.

[CIT0027] Green A. R, Marsden C, Fone K (2008). MDMA as a clinical tool: A note of caution. A response to Sessa and Nutt. Journal of Psychopharmacology.

[CIT0028] Green A. R, Mechan A. O, Elliott J. M, O'Shea E, Colado M. I (2003). The pharmacology and clinical pharmacology of 3,4-methylenedioxymethamphetamine (MDMA, “ecstasy”). Pharmacological Reviews.

[CIT0029] Guastella A, Dadds M, Lovibond P, Mitchell P, Richardson R (2007). A randomized controlled trial of the effect of d-cycloserine on exposure therapy for spider fear. Journal of Psychiatric Research.

[CIT0030] Hardman J, Limbird L (2001). Goodman & Gilman's pharmacological basis of therapeutics.

[CIT0031] Hofmann S. G, Meuret A. E, Smits J. A. J, Simon N. M, Pollack M. H, Eisenmenger K (2006). Augmentation of exposure therapy with D-cycloserine for social anxiety disorder. Archives of General Psychiatry.

[CIT0032] Hysek C. M, Domes G, Liechti M. E (2012). MDMA enhances “mind reading” of positive emotions and impairs “mind reading” of negative emotions. Psychopharmacology.

[CIT0033] Hysek C. M, Simmler L. D, Nicola V. G, Vischer N, Donzelli M, Krähenbühl S (2012). Duloxetine inhibits effects of MDMA (“Ecstasy”) and in humans in a randomized placebo-controlled laboratory study. PLoS ONE.

[CIT0034] Johansen P. O, Krebs T. S (2009). How could MDMA (ecstasy) help anxiety disorders? A neurobiological rationale. Journal of Psychopharmacology.

[CIT0035] Kehle-Forbes S. M, Polusny M. A, MacDonald R, Murdoch M, Meis L. A, Wilt T. J (2012). A systematic review of the efficacy of adding nonexposure components to exposure therapy for posttraumatic stress disorder. Psychological Trauma: Theory, Research, Practice, and Policy.

[CIT0036] Kessler R. C, Berglund P, Demler O, Jin R, Merikangas K. R, Walters E. E (2005). Lifetime prevalence and age-of-onset distributions of DSM-IV disorders in the national comorbidity survey replication. Archives of General Psychiatry.

[CIT0037] Kindt M, Soeter M (2013). Reconsolidation in a human fear conditioning study: A test of extinction as updating mechanism. Biological Psychology.

[CIT0038] Kindt M, Soeter M, Vervliet B (2009). Beyond extinction: Erasing human fear responses and preventing the return of fear. Nature Neuroscience.

[CIT0039] Kolbrich E. A, Goodwin R. S, Gorelick D. A, Hayes R. J, Stein E. A, Huestis M. A (2008). Physiological and subjective responses to controlled oral 3,4-methylenedioxymethamphetamine administration. Journal of Clinical Psychopharmacology.

[CIT0040] Kushner M, Kim S, Donahue C, Thuras P, Adson D, Kotlyar M (2007). D-cycloserine augmented exposure therapy for obsessive-compulsive disorder. Biological Psychiatry.

[CIT0041] Ledgerwood L, Richardson R, Cranney J (2003). Effects of D-cycloserine on extinction of conditioned freezing. Behavioral Neuroscience.

[CIT0042] LeDoux J. E (1995). Emotion: Clues from the brain. Annual Review of Psychology.

[CIT0043] Liechti M. E, Baumann C, Gamma A, Vollenweider F. X (2000). Acute psychological effects of 3,4-methylenedioxymethamphetamine (MDMA, “Ecstasy”) are attenuated by the serotonin uptake inhibitor citalopram. Neuropsychopharmacology.

[CIT0044] Litz B. T, Salters-Pedneault K, Steenkamp M. M, Hermos J. A, Bryant R. A, Otto M. W (2012). A randomized placebo-controlled trial of d-cycloserine and exposure therapy for posttraumatic stress disorder. Journal of Psychiatric Research.

[CIT0045] Lonergan M. H, Olivera-Figueroa L. A, Pitman R. K, Brunet A (2012). Propranolol's effects on the consolidation and reconsolidation of long-term emotional memory in healthy participants: A meta-analysis. Journal of Psychiatry and Neuroscience.

[CIT0046] MacDonald E, Dadds M. R, Brennan J. L, Williams K, Levy F, Cauchi A. J (2011). A review of safety, side-effects and subjective reactions to intranasal oxytocin in human research. Psychoneuroendocrinology.

[CIT0047] Milekic M. H, Alberini C. M (2002). Temporally graded requirement for protein synthesis following memory reactivation. Neuron.

[CIT0048] Mills K. L, Teesson M, Back S. E, Brady K. T, Baker A. L, Hopwood S (2012). Integrated exposure-based therapy for co-occurring posttraumatic stress disorder and substance dependence: A randomized controlled trial. JAMA.

[CIT0049] Mills K. L, Teesson M, Ross J, Peters L (2006). Trauma, PTSD, and substance use disorders: Findings from the Australian National Survey of Mental Health and Well-Being. American Journal of Psychiatry.

[CIT0050] Mithoefer M. C, Wagner M. T, Mithoefer A. T, Jerome L, Doblin R (2011). The safety and efficacy of {+/−}3,4-methylenedioxymethamphetamine-assisted psychotherapy in subjects with chronic, treatment-resistant posttraumatic stress disorder: The first randomized controlled pilot study. Journal of Psychopharmacology.

[CIT0051] Mithoefer M. C, Wagner M. T, Mithoefer A. T, Jerome L, Martin S. F, Yazar-Klosinski B (2013). Durability of improvement in post-traumatic stress disorder symptoms and absence of harmful effects or drug dependency after 3,4-methylenedioxymethamphetamine-assisted psychotherapy: A prospective long-term follow-up study. Journal of Psychopharmacology.

[CIT0052] Norberg M. M, Krystal J. H, Tolin D. F (2008). A meta-analysis of D-cycloserine and the facilitation of fear extinction and exposure therapy. Biological Psychiatry.

[CIT0053] Oehen P, Traber R, Widmer V, Schnyder U (2013). A randomized, controlled pilot study of MDMA (±3,4-Methylenedioxymethamphetamine)-assisted psychotherapy for treatment of resistant, chronic Post-Traumatic Stress Disorder (PTSD). Journal of Psychopharmacology.

[CIT0054] Olff M, Langeland W, Witteveen A, Denys D (2010). A psychobiological rationale for oxytocin in the treatment of posttraumatic stress disorder. CNS Spectrum.

[CIT0055] Otto M. W, Tolin D. F, Simon N. M, Pearlson G. D, Basden S, Meunier S. A (2010). Efficacy of D-cycloserine for enhancing response to cognitive-behavior therapy for panic disorder. Biological Psychiatry.

[CIT0056] Parsons R. G, Ressler K. J (2013). Implications of memory modulation for post-traumatic stress and fear disorders. Nature Neuroscience.

[CIT0057] Phelps E. A, Delgado M. R, Nearing K. I, LeDoux J. E (2004). Extinction learning in humans: Role of the amygdala and vmPFC. Neuron.

[CIT0058] Powers M. B, Halpern J. M, Ferenschak M. P, Gillihan S. J, Foa E. B (2010). A meta-analytic review of prolonged exposure for posttraumatic stress disorder. Clinical Psychology Review.

[CIT0059] Resick P. A, Galovski T. E, O'Brien Uhlmansiek M, Scher C. D, Clum G. A, Young-Xu Y (2008). A randomized clinical trial to dismantle components of cognitive processing therapy for posttraumatic stress disorder in female victims of interpersonal violence. Journal of Consulting and Clinical Psychology.

[CIT0060] Ressler K. J, Rothbaum B. O, Tannenbaum L, Anderson P, Graap K, Zimand E (2004). Cognitive enhancers as adjuncts to psychotherapy: Use of D-cycloserine in phobic individuals to facilitate extinction of fear. Archives of General Psychiatry.

[CIT0061] Richardson R, Ledgerwood L, Cranney J (2004). Facilitation of fear extinction by D-cycloserine: Theoretical and clinical implications. Learning & Memory.

[CIT0062] Rothbaum B. O, Cahill S. P, Foa E. B, Davidson J. R. T, Compton J, Connor K. M (2006). Augmentation of sertraline with prolonged exposure in the treatment of posttraumatic stress disorder. Journal of Traumatic Stress.

[CIT0063] Schneier F. R, Neria Y, Pavlicova M, Hembree E, Suh E. J, Amsel L (2012). Combined prolonged exposure therapy and paroxetine for PTSD related to the World Trade Center attack: A randomized controlled trial. American Journal of Psychiatry.

[CIT0064] Schnurr P. P, Friedman M. J, Engel C. C, Foa E. B, Shea M, Chow B. K (2007). Cognitive behavioral therapy for posttraumatic stress disorder in women: A randomized controlled trial. JAMA.

[CIT0065] Sessa B (2008). Is there a case for MDMA-assisted psychotherapy in the UK? (vol 21, pg 220, 2007). Journal of Psychopharmacology.

[CIT0066] Siegmund A, Golfels F, Finck C, Halisch A, Räth D, Plag J (2011). D-cycloserine does not improve but might slightly speed up the outcome of in-vivo exposure therapy in patients with severe agoraphobia and panic disorder in a randomized double blind clinical trial. Journal of Psychiatric Research.

[CIT0067] Simon N. M, Connor K. M, Lang A. J, Rauch S. A. M, Krulewicz S, LeBeau R. T (2008). Paroxetine CR augmentation for posttraumatic stress disorder refractory to prolonged exposure therapy. Journal of Clinical Psychiatry.

[CIT0068] Smits J. A. J, Rosenfield D, Otto M. W, Powers M. B, Hofmann S. G, Telch M. J (2013). D-cycloserine enhancement of fear extinction is specific to successful exposure sessions: evidence from the treatment of height Phobia. Biological Psychiatry.

[CIT0069] Soravia L. M, Heinrichs M, Aerni A, Maroni C, Schelling G, Ehlert U (2006). Glucocorticoids reduce phobic fear in humans. Proceedings of the National Academy of Sciences of the United States of America.

[CIT0070] Storch E. A, Merlo L. J, Bengtson M, Murphy T. K, Lewis M. H, Yang M. C (2007). D-cycloserine does not enhance exposure-response prevention therapy in obsessive-compulsive disorder. International Clinical Psychopharmacology.

[CIT0071] Suris A, North C, Adinoff B, Powell C. M, Greene R (2010). Effects of exogenous glucocorticoid on combat-related PTSD symptoms. Annals of Clinical Psychiatry.

[CIT0072] Suzuki A, Josselyn S. A, Frankland P. W, Masushige S, Silva A. J, Kida S (2004). Memory reconsolidation and extinction have distinct temporal and biochemical signatures. The Journal of Neuroscience.

[CIT0073] Tart C. D, Handelsman P. R, Deboer L. B, Rosenfield D, Pollack M. H, Hofmann S. G (2013). Augmentation of exposure therapy with post-session administration of d-cycloserine. Journal of Psychiatric Research.

[CIT0074] Thompson M. R, Callaghan P. D, Hunt G. E, Cornish J. L, McGregor I. S (2007). A role for oxytocin and 5-HT(1A) receptors in the prosocial effects of 3,4 methylenedioxymethamphetamine (“ecstasy”). Neuroscience.

[CIT0075] van Minnen A, Hagenaars M (2002). Fear activation and habituation patterns as early process predictors of response to prolonged exposure treatment in PTSD. Journal of Traumatic Stress.

[CIT0076] van Minnen A, Harned M. S, Zoellner L, Mills K (2012). Examining potential contraindications for prolonged exposure therapy for PTSD. European Journal of Psychotraumatology.

[CIT0077] Walker D. L, Ressler K. J, Lu K. T, Davis M (2002). Facilitation of conditioned fear extinction by systemic administration or intra-amygdala infusions of D-cycloserine as assessed with fear-potentiated startle in rats. Journal of Neuroscience.

[CIT0078] Werner-Seidler A, Richardson R (2007). Effects of D-cycloserine on extinction: Consequences of prior exposure to imipramine. Biological Psychiatry.

[CIT0079] Yehuda R, Bierer L. M, Pratchett L, Malowney M (2010). Glucocorticoid augmentation of prolonged exposure therapy: Rationale and case report. European Journal of Psychotraumatology.

